# Supplementary material for: Exposure–Response Relationships for Toceranib in Dogs with Solid Tumors: A Pilot Study
Source: Animals (Basel). 2025 Apr 2;15(7):1025. doi: 10.3390/ani15071025 (PMC11988034; doi:10.3390/ani15071025)
Supplement: Supplementary file 1 [file animals-15-01025-s001.zip › animals-3499755-supplementary.pdf]

Supplementary Materials

Table S1. Adverse events observed in dogs treated with toceranib.

Category	Grade 1	Grade 2	Grade 3	Grade 4	Grade 5
Constitutional					
Lethargy	2				
Gastrointestinal					
Inappetence		2			
Anorexia					
Vomiting					
Diarrhea		1	1		
Hematologic					
Anemia					
Neutropenia	2				
Thrombocytopenia					
Biochemical					
Increased ALT					
Increased AST	2				
Increased ALP					
Renal					
Proteinuria			1		
Cardiovascular					
Hypertension					
Total	6	3	1	0	0

Note: Adverse events were evaluated according to the Veterinary Cooperative Oncology Group-Common Terminology Criteria for Adverse Events (VCOG-CTCAE v2.0). Abbreviation: ALT, alanine aminotransferase; AST, aspartate aminotransferase; ALP, alkaline phosphatase.
